# Anaplastic Large-Cell Lymphoma in a Child with Type I Diabetes and Unrecognised Coeliac Disease

**DOI:** 10.1155/2012/269689

**Published:** 2012-10-03

**Authors:** Jemima Sharp, Barry Pizer, George Kokai, Marcus K. H. Auth

**Affiliations:** ^1^Department of Paediatric Gastroenterology, Alder Hey Children's NHS Foundation Trust, Eaton Road, Liverpool L12 2AP, UK; ^2^Department of Paediatric Oncology, Alder Hey Children's NHS Foundation Trust, Eaton Road, Liverpool L12 2AP, UK; ^3^Department of Paediatric Pathology, Alder Hey Children's NHS Foundation Trust, Eaton Road, Liverpool L12 2AP, UK

## Abstract

Screening for coeliac disease is recommended for children from certain risk groups, with implications for diagnostic procedures and dietetic management. The risk of a malignant complication in untreated coeliac disease is not considered high in children. We present the case of a girl with type I diabetes who developed weight loss, fatigue, and inguinal lymphadenopathy. Four years before, when she was asymptomatic, a screening coeliac tTG test was positive, but gluten was not eliminated from her diet. Based on clinical examination, a duodenal biopsy, and an inguinal lymph node biopsy were performed, which confirmed both coeliac disease and an anaplastic large-cell lymphoma. HLA-typing demonstrated that she was homozygous for HLA-DQ8, which is associated with higher risk for celiac disease, more severe gluten sensitivity, and diabetes susceptibility. She responded well to chemotherapy and has been in remission for over 4 years. She remains on a gluten-free diet. This is the first case reporting the association of coeliac disease, type I diabetes, and anaplastic large-cell lymphoma in childhood. The case highlights the malignancy risk in a genetically predisposed individual, and the possible role of a perpetuated immunologic response by prolonged gluten exposure.

## 1. Introduction

The association between coeliac disease (CD) and lymphoma has been reported for more than two decades, but the clinical significance has remained a controversial topic [1]. The most frequently occurring malignancy associated with CD is enteropathy-type T-cell lymphoma (ETTL) of the upper small intestine. However CD is now known to be associated with other non-Hodgkin lymphomas of both B- and T-cell type, in either gut or other primary sites [1]. Most of this evidence has come from studies in adult patients.

In the literature, only few cases of malignancy occurring in children with CD have been described, such as the case reported by Stenhammar and Masreliez [2]. In 2001, Schweizer et al. surveyed members of the European Society for Paediatric Gastroenterology, Hepatology, and Nutrition. Over a 10 year period, they identified 21 new cases of malignancy in children with CD. An increased number of small bowel and thyroid malignancies were reported, suggesting an association with CD [3]. However, the incidence of malignancy in children with CD is not known and is probably underreported. Here, we present the first case of anaplastic large cell lymphoma (ALCL) in a young girl with CD.

## 2. Case Report

A 10-year-old girl was referred by her local hospital to a tertiary centre for an upper GI endoscopy and biopsy. The girl had been diagnosed with insulin-dependent diabetes mellitus (IDDM) 4 years previously, remaining clinically well until four months prior to referral, when she presented with a history of weight loss (approximately 10% of her weight). During an assessment at her local hospital it emerged that an antitissue transglutaminase antibody (tTG) blood test (a screening test for CD) performed at the initial presentation of her IDDM, had been positive. Now presenting with weight loss, she was referred urgently for further investigation.

The patient had no overt gastrointestinal symptoms, but interestingly did have a 4-month history of lymphadenopathy. She also had a recent infected insect bite on her arm, lived with cats and had had contact with a family member with suspected tuberculosis (TB). The lymphadenopathy, located in her left groin, was nontender, varied in size, and had improved when treated with antibiotics at her local hospital. An ultrasound scan performed at her local hospital was suggestive of infected lymph nodes. Clinically the patient had no other palpable nodes, and there was no hepatosplenomegaly. The initial differential diagnosis included borrelia, bartonella, and TB. 

The case was complicated by the patient's significant needle phobia and it was decided that all haematological investigations would be performed whilst the patient had an upper GI endoscopy under general anaesthesia. An ultrasound scan was repeated, confirming the enlarged nodes in her groin, and also demonstrating para-aortic lymphadenopathy. A subsequent CT scan also demonstrated cervical lymphadenopathy as well as the abdominal lymphadenopathy, and at this stage a diagnosis of lymphoma was considered.

During the general anaesthetic, a groin lymph node was removed for histopathology. Blood taken at this time showed normal haemoglobin and white cell count, but a mildly elevated platelet count (600 × 10^9^/L) and inflammatory markers (CRP 29 mg/L and ESR 36 mm/hour). A peripheral blood film was normal. A repeat tTG was initially mildly elevated at 16 u/mL and subsequently negative at 0.7 u/mL (normal range 0–7 u/mL).

**Figure 1:**
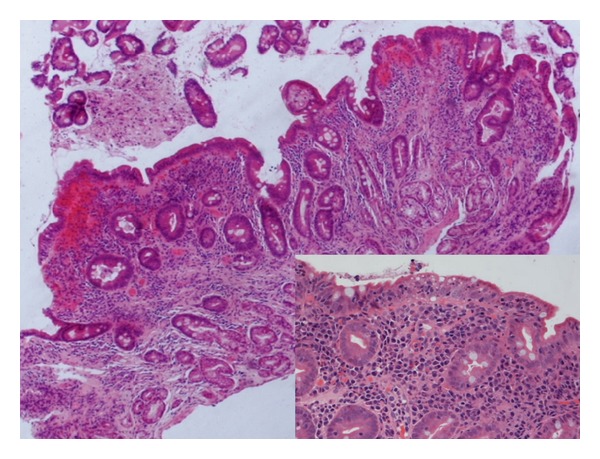
Duodenal biopsy following four year history of positive tTG-antibodies: Significant villous atrophy, hyperplastic crypts, dense mononuclear infiltrate in duodenal mucosa (Marsh 3a-b) (HE, ×40 original magnification). Pseudostratified enterocytes, increased number of intra-epithelial lymphocytes/IELs and loss of mucus cells (inset, HE, ×120 original magnification).

Duodenal biopsy of small bowel confirmed the diagnosis of CD with villous atrophy, crypt hyperplasia, and increased intraepithelial lymphocytes (Marsh 3a-b according to the modified classification, Figure 1), and so the patient was commenced on a gluten-free diet.

**Figure 2:**
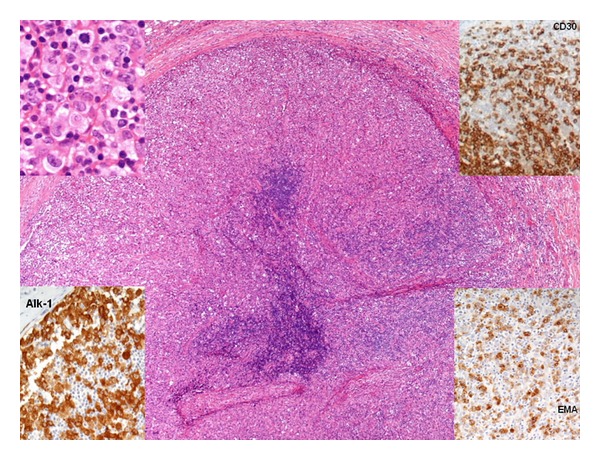
Inguinal lymph node biopsy illustrating anaplastic large cell lymphoma (ALCL): Lymph node replaced by nodules of proliferating large, pale, anaplastic-looking tumour cells of lymphoid origin (H&E, ×80 original magnification) positive with immuno-labelling for Alk-1, CD30 and EMA (insets; ×150 original magnification).

The lymph node biopsy showed morphology and immunohistochemistry of an ALCL (Figure 2). 

HLA-typing demonstrated that she was homozygous for HLA-DQ8, which is associated with higher risk for coeliac disease, more severe gluten sensitivity, and diabetes susceptibility. Treatment was given according to the European ALCL 99 protocol, with six courses of multiagent chemotherapy. The patient responded well to treatment and 4 years following chemotherapy remained in clinical and radiological remission. 

## 3. Discussion

The clinical presentation of CD is changing. Only a proportion of children present classically with severe gastrointestinal disturbance, poor growth, weight loss, and malnutrition. The majority of people with CD have mild symptoms or are asymptomatic, a proportion of these being identified through targeted screening. Mass screening is not currently performed in the UK. Among patients recommended for screening include those diagnosed with IDDM, who are known to have an increased risk of CD [4], due to shared HLA susceptibility for DQ2 and DQ8. 

Diabetic children with CD often have no obvious clinical symptoms of their CD. Therefore, the screening tests may escape the attention of clinicians focussing on management of IDDM.

But should we be concerned by a delay in diagnosis especially if the child has no overt gastrointestinal symptoms? CD is known to be associated with anaemia, short stature, delayed puberty, arthralgia, and most seriously certain types of lymphoma [1, 4]. Although the malignancy most commonly associated with CD is ETTL, evidence is emerging that the overall risk of other lymphomas, including extra-intestinal disease is increased in patients with CD [1]. As well as CD, our patient was diagnosed with an ALCL, a T-cell non-Hodgkin lymphoma, which arose outside the GI tract. ALCL is characterised by larger-sized T-cells, typically immunoreactive for anaplastic lymphoma kinase protein, and requires treatment according to aggressive lymphoma guidelines. A pooled analysis by Smedby et al. has shown that in the adult population, ALCL is 24 times more common in those with CD [5]. Until now, in children the association of ALCL with CD has not been reported. The 2001 paediatric survey reported, amongst its 21 previously unreported malignancies in patients with CD, 6 cases of non-Hodgkin lymphoma. The 4 cases of NHL that arose in the small bowel occurred in children not on a gluten-free diet [3]. 

Evidence suggests that in adult patients, steps can be taken to prevent the development of lymphoma. In adult patients diagnosed with CD that adhere to a strict gluten-free diet for five consecutive years, the risk of malignancy falls to that of the general population [6], whereas untreated, the risk of malignancy remains. 

In this case, the question remains as to whether or not this lymphoma was associated with the undiagnosed CD, and whether or not earlier detection of the CD (which should have occurred with screening) and instigation of a gluten-free diet may have prevented this outcome. This patient was subsequently tested positive for two DQ-specific serotype patterns; DQ8/DQ8 carries a much higher risk for CD and significantly more severe forms of gluten sensitivity [7, 8]. This association can be explained by the fact that gluten peptides can be presented in and HLA-DQ8 molecules on antigen presenting cells. Already 1 microgram of gluten per day is enough to elicit a T-cell response, and a normal diet contains about 10–15 grams gluten per day. The constant antigen exposure triggers a continuous polyclonal T-cell response, in which T-cells with the highest affinity to multiple epitopes survive. It has been proposed that additional genetic polymorphisms may influence T-cell reactivity, in which ultimately aberrant T-cells can continue to proliferate into a lymphoma [9]. 

The case also demonstrates the importance of fully investigating children when the clinical presentation does not fit the expected pattern. The weight loss with which the child presented was assumed to be related to the suspected CD but the associated lymphadenopathy was an unusual feature, and not obviously explained by a diagnosis of CD.

We believe this to be the first reported case of anaplastic large-cell lymphoma arising in a child with CD and IDDM. The case highlights the malignancy risk in genetically predisposed individual already in childhood, and the possible role of a perpetuated immunologic response by prolonged gluten exposure.
